# Disulfide Bonds within the C2 Domain of RAGE Play Key Roles in Its Dimerization and Biogenesis

**DOI:** 10.1371/journal.pone.0050736

**Published:** 2012-12-17

**Authors:** Wen Wei, Leonie Lampe, Sungha Park, Bhavana S. Vangara, Geoffrey S. Waldo, Stephanie Cabantous, Sarah S. Subaran, Dongmei Yang, Edward G. Lakatta, Li Lin

**Affiliations:** 1 Laboratory of Cardiovascular Science, National Institute on Aging, Baltimore, Maryland, United States of America; 2 Bioscience Division, MS-M888, Los Alamos National Laboratory, Los Alamos, New Mexico, United States of America; Carl-Gustav Carus Technical University-Dresden, Germany

## Abstract

**Background:**

The receptor for advanced glycation end products (RAGE) on the cell surface transmits inflammatory signals. A member of the immunoglobulin superfamily, RAGE possesses the V, C1, and C2 ectodomains that collectively constitute the receptor's extracellular structure. However, the molecular mechanism of RAGE biogenesis remains unclear, impeding efforts to control RAGE signaling through cellular regulation.

**Methodology and Result:**

We used co-immunoprecipitation and crossing-linking to study RAGE oligomerization and found that RAGE forms dimer-based oligomers. Via non-reducing SDS-polyacrylamide gel electrophoresis and mutagenesis, we found that cysteines 259 and 301 within the C2 domain form intermolecular disulfide bonds. Using a modified tripartite split GFP complementation strategy and confocal microscopy, we also found that RAGE dimerization occurs in the endoplasmic reticulum (ER), and that RAGE mutant molecules without the double disulfide bridges are unstable, and are subjected to the ER-associated degradation.

**Conclusion:**

Disulfide bond-mediated RAGE dimerization in the ER is the critical step of RAGE biogenesis. Without formation of intermolecular disulfide bonds in the C2 region, RAGE fails to reach cell surface.

**Significance:**

This is the first report of RAGE intermolecular disulfide bond.

## Introduction

RAGE was initially identified as a receptor for advanced glycation end products (AGE) [1], which are generated via non-enzymatic crosslinking of carbohydrates to proteins and other biological molecules [2]. Since then, other ligands for RAGE have been discovered including chromatin binding protein HMGB1 (high-mobility group box 1), s100 family of small calcium binding peptides, amyloid β protein, and phosphatidylserine [3]–[6], making RAGE one of the pattern recognizing receptors that participate in innate immunity [7], [8]. In addition, RAGE also functions as an adhesion molecule on the cell surface of neutrophiles, enhancing its recruitment to vascular endothelial cells during inflammation [9]. Similar to the Toll-like receptors (TLRs), engagement of RAGE by its ligands triggers several intracellular signaling programs including NF-κB and Erk1/2 transcription pathways, leading to inflammation [10]–[12]. However, unlike ligands for TLRs, which are mainly derived from exogenous pathogens, RAGE ligands are generated either endogenously, or are derived from the diet [13], [14]. RAGE-associated signaling, therefore, appears to participate mainly in pathophysiological events such as inflammation-related tissue remodeling and maladaptation, and has been implicated in atherogenesis, diabetes, and Alzheimer's disease [11], [15], [16]. Recent reports have also shown that RAGE may be involved in immune defense mechanisms [17]–[19]. Despite its significant roles in pathogenesis and inflammation, the signaling mechanism of RAGE remains elusive, and cytosolic factors that relay the cell surface signals to specific cellular programs have not been elucidated [8], [12], [16].

The constituent of a receptor complex on the cell surface, including the oligomeric status of the receptor within the complex, is a crucial starting point of the signal relay. To date, neither RAGE complex, nor its oligomeric status on the cell surface have been clearly defined. Earlier studies using fluorescence resonance energy transfer assays demonstrated that RAGE forms homo-dimers and perhaps higher-order homo-oligomers on the cell surface in a ligand-independent manner [20]. However, the structural elements responsible for RAGE oligomerization have not been elucidated. More importantly, the functional impacts of this structural feature have not been realized.

We report here that RAGE forms constitutive homo-dimers via intermolecular disulfide bonds by cysteine residues 259 and 301 within the C2 ectodomain of the receptor. Converting the two cysteines to serines significantly reduces RAGE expression on the cell surface. Although RAGE molecules containing either or both cysteine mutations can form unstable dimers *in vivo* via non-covalent bonds, these unstable dimers, instead of reaching the cell surface, are diverted from the ER into the cytoplasm, deglycosylated, and subsequently degraded via the ubiquitin-proteasome pathway. This suggests that the disulfide bridge structure in the C2 region of RAGE serves as an inherent hallmark discerned by the cellular quality control system in the ER. Using a novel tripartite split green fluorescence protein (GFP) complementation analysis that renders observation of receptor dynamics in the ER, we also established that RAGE dimerization occurs in the ER. Revealing the molecular mechanism of RAGE dimerization and its biological implications should ameliorate our understanding of cellular regulation of RAGE biogenesis, and provide a starting point to further intercede RAGE signaling.

## Materials and Methods

### Cell culture and transfection

CHO-CD14 cells were grown in RPMI 1640 medium supplemented with 10% fetal calf serum (FCS) as described previously [21]. HeLa cells were grown in Dulbercco's modified eagle medium (DMEM) containing 10% FCS. Cell transfection was performed with Invitrogen Lipofectamine or Lipofectamine 2000 as described previously [22].

### Electrophoresis conditions

To achieve a non-reducing condition, the reducing reagent (50 mM dithiothreitol, DTT) was omitted from the loading buffer. Samples of both reducing and non-reducing conditions were mixed with loading buffer containing 2% lithium dodecyl sulfate (LDS) and heated at 80°C for 10 min before being resolved on Invitrogen NuPAGE SDS 4–12% Bis-Tris gel. For native PAGE, purified sRAGE were mixed with loading buffer and resolved with Invitrogen Blue Native (BN) 4–16% Bis-Tris gel.

### Immunoblotting, immunoprecipitation and antibodies

Immunoblotting (IB) and immunoprecipitation (IP) were performed as described previously [22]. For multiple IBs on the same blot, the membrane was washed with Invitrogen blot wash solution, and incubated with Restore Western blot stripping buffer (Thermo Scientific) at room temperature for 30 min before being subject to the next IB. Mouse anti-FLAG antibodies (M2, and M2 horseradish peroxidase conjugate) and mouse anti-β-actin antibodies (AC-40) were from Sigma-Aldrich Company; mouse anti-T7 antibodies were purchased from Novagen-EMD; rabbit anti-RAGE (H-300), anti-calnexin (H-70), anti-calregulin (H-170), and anti-NF-κB p50 (NLS) antibodies were products of Santa Cruz Biotechnology; rabbit anti-GFP antibodies were from Invitrogen; and rat anti-HA antibodies (3F10, horseradish peroxidase conjugate) were from Roche Applied Science.

### Preparation of unfractionated and fractionated cell membrane extract

Preparation of unfractionated cell membrane extracts was as previously described [22]. For preparation of crude cell membrane fraction, the transfected cells were washed twice with 1× phosphate buffered saline (PBS), and incubated with the swelling buffer (0.2 mM EDTA, 10 mM Tris-HCl buffer pH 7.5, 1 mM DTT, 1 mM phenylmethanesulphonylfluoride, and protease inhibitor cocktail from Sigma-Aldrich) on ice for 30 min. The cells were then scraped from the culture plates and sonicated for 3 cycles with 10 sec each in ice. The lysates were centrifuged with 1,500× *g* at 4°C for 10 min to obtain nuclear-free supernatants and 1 M of Na_2_CO_3_ was added to a final concentration of 0.2 M. The supernatants were then centrifuged with 45,000× *g* at 4°C for 30 min, and pellets were carefully washed with 1× PBS. The crude membrane pellets obtained are either used for further analysis or stored at −80°C.

### Mouse lung preparation

The lungs were isolated from the wild-type (C57BL/6) and RAGE knockout (KO) mice, according to the approved animal care protocol and NIH guidelines. After mincing to small pieces, the lung tissues were washed with ice cold 1× PBS twice, transferred to 10 mM Tris, pH 7.5, 0.2 mM EDTA buffer containing proteases inhibitors and 20 mM *N*-Ethylmaleimide, and homogenized with a ploytron homoginizer. The homogenized lung tissues were left on ice for 30 min and filtered through 3 layers of cheesecloth. The crude membranes were further extracted as described. This study was carried out in strict accordance with the recommendations in the Guide for the Care and Use of Laboratory Animals of the National Institute on Aging, NIH (approval number: 418-LCS-2012).

### Crosslinking reactions

For direct crosslinking of transfected CHO-CD14 cells in 35 mm culture plates, the cells were washed twice with 1× PBS, pH 8.0. Crosslinking reactions were conducted in the same buffer containing 2.5 mM BS^3^ (Thermo Scientific) at room temperature for 30 min on a shaker, and terminated with Tris-HCl, pH 7.5 (final concentration: 20 mM) for 15 min. Cells were then washed, lysed, and membrane extracts were prepared. The lysates were resolved with SDS 3–8% NuPAGE Tris-acetate gel (Invitrogen) for immunoblotting. For crosslinking of membrane extracts, 1 mM of BS^3^ was used and the reaction was conducted with the same conditions.

### Construction of plasmids used in the studies

T7- and FLAG-tagged RAGE plasmids as well as membrane-targeting expression vectors have been described previously [22]. These vectors ensure that the proteins of interest are targeted to the ER, and subsequently expressed on the cell surface. RAGE deletion mutants were constructed by ligating PCR fragments with primers flanking the deleted sequences, using RAGE (WT) as the template. RAGE cysteine-to-serine mutants were generated with Stratagene Quickchange mutagenesis kit, according to the manufacturer's instruction using relevant primers that carry mutated encoding nucleotides. All mutants were nucleotide-sequenced and confirmed.

For plasmids used in split GFP tripartite complementation analysis, we first constructed s10- and s11-tagged (at NH_2_ –terminus) membrane-targeting expression vectors by modifying previously published vectors [22], and subcloned WT and mutant RAGE into these vectors. For the s10-tagged vector, the subcloned target protein is also tagged with the FLAG epitope tag at its COOH-terminus, whereas in the s11-tagged vector, the target protein is tagged at its COOH- terminus with the T7 tag.

Signalpep-GFPs1-9 and signalpep-s11-p50 were generated by subcloning the relative target cDNA sequences into the membrane-targeting expression vectors [22].

### Immunocytochemistry and confocal microscopy

HeLa cells seeded on glass coverslips were transfected with Flag-tagged RAGE or RAGE mutants, and, after overnight incubation, fixed with 4% paraformaldhyde at room temperature for 15 min. Cells were then permeabilized with 0.2% Triton X-100 in 1× PBS for 15 min followed with 1 h blocking in 1% bovine serum albumin (BSA) in 1× PBS. After blocking, cells were incubated with anti-FLAG monoclonal antibodies and anti-calnexin antibodies (1∶250 of each in 1% BSA/1× PBS) for 2 h at room temperature. The cells were then incubated with donkey anti-mouse IgG conjugated with Alexa Fluor 546 and goat anti-rabbit IgG conjugated with Alexa Fluor 488 (Invitrogen, 1∶1000 of each in 1% BSA/1× PBS) for 1 h at room temperature. The coverslips were mounted with ProLong Gold antifade reagent containing 4′-6-diamidino-2-phenylindole (DAPI) (Invitrogen) at room temperature for 48 h with cover to prevent photobleach. Images were obtained using a Zeiss LSM 510 Meta confocal microscope with a Plan-Neofluar 40×/1.3 oil DIC objective. For tripartite complementation analysis with confocal, anti-calregulin antibodies (1∶100) and donkey anti-rabbit IgG conjugated with Alexa Fluo 546 (Invitrogen, 1∶1000) were used. Following the secondary antibody incubation, cells were washed 3 times with 1× PBS and incubated with 1 µM To-Pro-3 iodide (642/661) (Invitrogen) at room temperature for 15 min followed by 3 times wash with 1× PBS.

### Split GFP tripartite complementation in the ER

Split GFP tripartite complementation has been described (http://www.lanl.gov/projects/gfp/P-P_interaction.shtml). CHO-CD 14 cells (2×10^5^) were seeded on 35 mm plates and transfected as described. After overnight incubation, transfected cells were washed once with 1× PBS and supplemented with fresh medium. Images were obtained using a Zeiss Axiovert 200 fluorescence microscope with a 10× objective. The cells were subsequently either lysed for IB to confirm the expression of the tripartite members, or treated with trypsin to obtain cell suspension for flow cytometric analyses.

### Cycloheximide chase

CHO-CD14 cells were seeded in individual 35 mm cell culture plates and transfected accordingly. Onto the next day, the cells were washed once with 1× PBS and replenished with fresh RPMI 1640 medium. Cycloheximide was added to the medium (200 µg/ml), and cells were incubated at 37°C cell culture incubator. At each time point, cells were lysed and processed for IB.

### Deglycosylation reactions

Deglycosylation of the *N*-link glycans of the target proteins was performed as previously described [22]. PNGase F was purchased from the New England Biolabs.

### Ubiquitination assays

CHO-CD 14 cells were co-transfected with RAGE/RAGE cysteine-to-serine mutants and an HA-ubiquitin construct. After overnight incubation, the cells were washed once with 1× PBS and replenished with fresh medium. The cells were then treated with the proteasome inhibitor MG 132 (10 µM, Calbiochem) for 3 h and lysed for co-IP analysis.

### Flow cytometric analyses

For GFP complementation assays, the transfected CHO-CD 14 cells were washed once with 1× PBS and treated with trypsin at 37°C for 2 min. RPMI 1640 medium was added to terminate the protease reaction, and the cell suspensions were centrifuged for 5 min with 300 rpm. The medium was then removed and cells resuspended in 1 ml 1× PBS and fixed with 1% paraformaldehyde. Flow cytometric analyses were performed with a FACSCanto (Becton Dickinson). Dead cells were excluded by forward light scatter and data were acquired. Untransfected cells were used to determine autofluorescence.

For cell surface staining, FLAG-tagged RAGE and RAGE mutants were transfected to CHO cells. After overnight incubation, the cells (10^6^) were washed with 1% BSA in PBS, and incubated with mouse anti-FLAG antibodies (Sigma, 1∶ 250 in 1% BSA) at 4°C for 30 min. After wash with 1% BSA, the cells were incubated with goat anti-mouse IgG conjugated with Alexa Fluor 488 (Invitrogen, 1∶1000 in 1% BSA) at 4°C for 30 min followed with washes and fixing. Untransfected cells were stained with the same antibodies and used as the negative control.

### Statistics

One-way analysis of variance (ANOVA) and Tukey post hoc analyses were used and the data were calculated as means ± standard errors of the means (SEM). *P*<0.05 was considered to be statistically significant.

## Results

### RAGE is oligomerized

**Figure 1:**
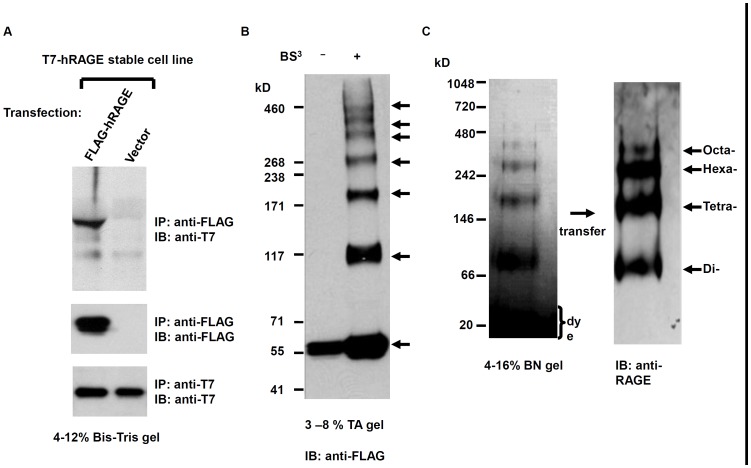
Oligomerization of RAGE. (**A**) Co-immunoprecipitation of differentially tagged RAGE. (**B**) Crosslinking of oligomeric RAGE. FLAG-tagged RAGE cDNA was tranfected to CHO-CD 14 cells and membrane fraction was crosslinked with BS^3^ and resolved with SDS-PAGE. Arrows indicate (starting from the bottom) resolved monomers, dimers, trimers, tetramers, pentamers, hexamers, and heptamers. (**C**) Human recombinant soluble RAGE resolved with Invitrogen blue native gel, and blotted with anti-RAGE antibodies. Arrows indicate (starting from the bottom) resolved dimers, tetramers, hexamers, and octamers.

To investigate the mechanism and biological consequence of RAGE oligomerization, we sought to first confirm the oligomerization status of RAGE via multilateral approaches using mammalian cell system. To perform co-IP studies, we transiently transfected FLAG tagged RAGE into a Chinese hamster ovary (CHO-CD14) cell line that was stably transfected with T7-tagged RAGE. Since the expression of tagged RAGE on the cell surface was previously confirmed [22], co-IP was performed directly with un-fractionated membrane extracts. As shown in Figure 1A, IP of transfected cell extracts with anti-FLAG antibodies followed by IB with anti-T7 antibodies on the precipitants resolved by SDS-PAGE demonstrated the formation of oligomer of RAGE. Crosslinking membrane fraction from transiently transfected CHO-CD14 cells with a non-cleavable and membrane-impermeable crosslinker, bis-sulfosuccinimidyl suberate (BS^3^), followed with IB on membrane extracts resolved by SDS-PAGE showed RAGE oligomeric ladders (Figure 1B). Since the crosslinking reaction was performed directly on cultured cells, or fractionated membrane preparations the observed higher-order RAGE oligomers reflect the receptor's configuration on the cell surface.

### A dimer is the underlining unit for higher-order RAGE oligomers

We also assessed oligomeric status of soluble RAGE (sRAGE), which lacks the transmembrane helix and the cytosolic portion, and is secreted into the extracellular milieu. Recombinant human sRAGE was expressed in CHO-CD14 cells, affinity-chromatographically purified, resolved with native PAGE, and then transferred to Immobilon membrane for IB. As shown in Figure 1C, sRAGE also forms oligomers. Interestingly, no monomeric sRAGE was detected on native PAGE, and sRAGE in the native condition appears to assemble into dimer-based oligomers (*i.e.* dimer, tetramer, hexamer, octamer). The observation of sRAGE oligomers in the native condition implies that a dimer is the underlying unit in the higher-order RAGE oligomers.

### Two cysteines within the C2 region form intermolecular disulfide bonds

**Figure 2:**
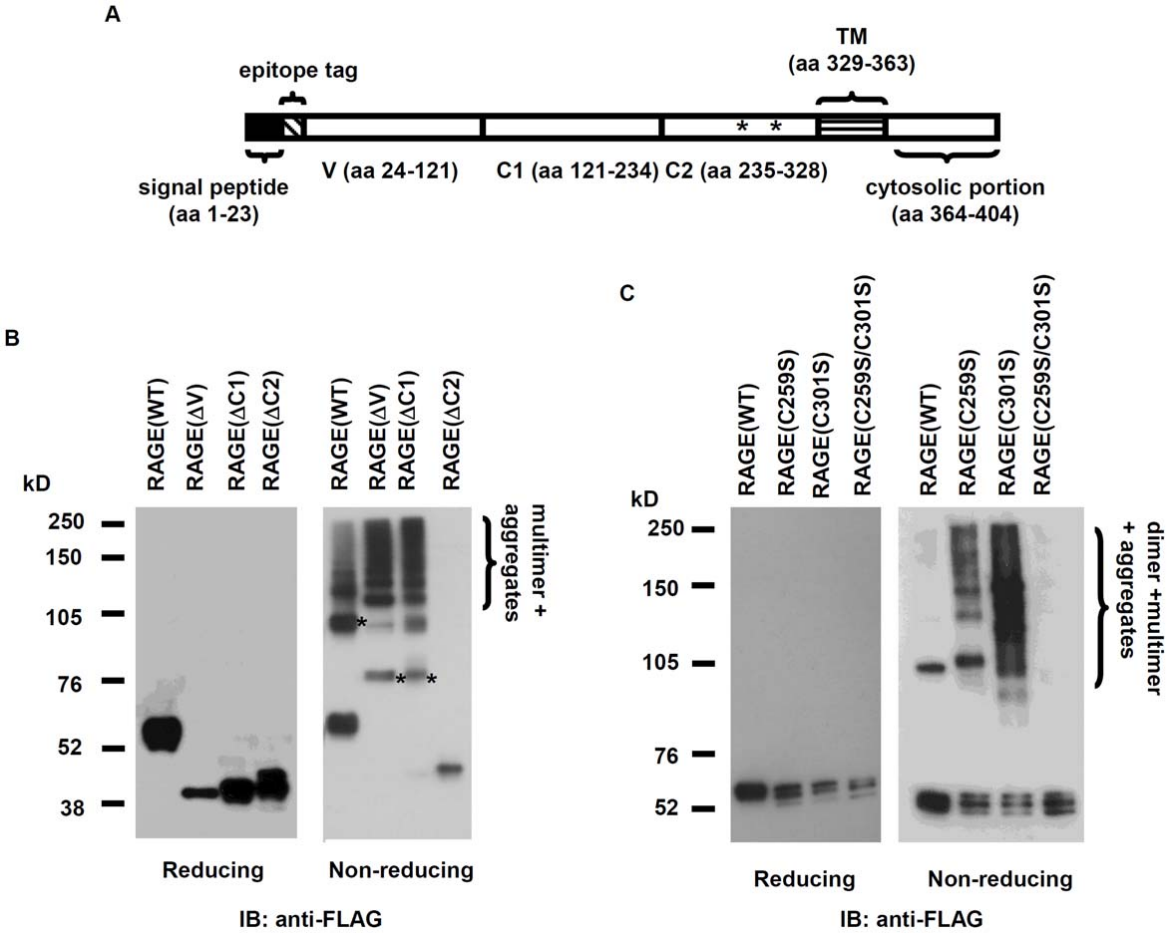
Intermolecular disulfide bonds contribute to the formation of RAGE dimers. (**A**) Schematic drawing of RAGE domains. aa: amino acids; * indicates C_259_ and C_301_; TM: transmembrane helix. (**B**) Identification of RAGE domain that is responsible for covalent-linked dimerization. About 5 µg of total membrane extracts was loaded. * indicate dimers of RAGE (WT) and deletion mutants. (**C**) RAGe C2 domain exhibits disulfide bond-mediated dimerization. * indicate dimers of RAGE(WT) and RAGE(C2). (**D**) Testing whether C_259_ and C_301_ are responsible for covalent-linked dimerization of RAGE. About 7.5 µg of total membrane extracts was loaded.

To identify the structural elements that contribute to RAGE dimerization, we made sequential domain deletions of the extracellular portion of the receptor (Figure 2A), and expressed these RAGE deletion mutants on the cell surface. Oligomeric proteins are sustained by both covalent and non-covalent bonds. We first tested whether covalent disulfide bonds are required for RAGE oligomerization. As shown in Figure 2B, while the wild-type (WT) RAGE and its deletion mutants that lack V (ΔV) or C1 (ΔC1) domain form oligomers in a non-reducing electrophoresis condition, the RAGE molecule without its C2 domain (ΔC2) is completely devoid of oligomers in this condition. This observation clearly indicates that disulfide bonds within C2 region contribute to RAGE dimerization. To test whether the observed covalent bond-mediated dimerization is indeed restricted within the C2 region, we also constructed C2 domain-only fragment (residues 235–404) and expressed this fragment on the cell surface. C2 domain exhibits disulfide bond-mediated dimers under non-reducing condition (Figure 2C). Of note, unlike the RAGR WT and domain deletions, C2 fragment under non-reducing condition showed monomers and dimers without additional aggregations, suggesting that disulfide bond-mediated dimerization within C2 region is rather specific, and that aggregations observed in the RAGE WT and deletion mutants are likely due to non-specific disulfide bond formation in the V and the C1 region. To assure that disulfide bond-mediated RAGE dimerization is not a cell-type specific phenomenon, we also performed the same studies in HeLa cells and obtained similar results (Supporting information Figure S1).

We next proceeded to convert the only two cysteine residues within C2 domain, C_259_ and C_301_, into serine residues, and tested oligmerization of RAGE carrying single or double cysteine mutations in the same non-reducing condition. Oligimerization is completely abolished *in vitro* when RAGE carries double cysteine mutations (C259S/C301S) (Figure 2D, right panel, right lane), suggesting that both C_259_ and C_301_ contribute to dimerization of RAGE.

Since disulfide bonds may form within or between molecules, it is possible that an intramolecular disulfide bridge between C_259_ and C_301_ generates a stable, low energy conformation that facilitates RAGE oligomerization via non-covalent forces. However, as shown in Figure 2D (right panel, middle two lanes), both RAGE(C259S) and RAGE(C301S) still form oligomers in a non-reducing condition, suggesting that disulfide bridges are formed between two RAGE molecules, and that the remaining single disulfide bridge in either RAGE(C259S) or RAGE(C301S) can still sustain a dimer. From these studies, we conclude that a RAGE dimeric structure is sustained by two intermolecular disulfide bonds between C_259_ and C_301_.

**Figure 3:**
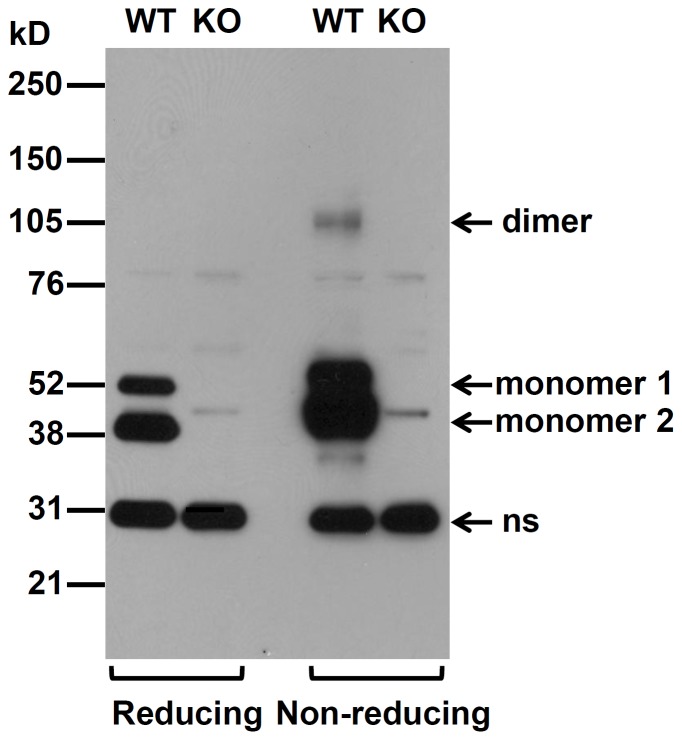
RAGE from mouse lung also exhibits disulfide-bond mediated dimeric structure. The lungs were isolated from both wild-type and RAGE(KO) mice and crude membrane fraction was prepared. The extracted membrane protein lysates (15 µg) were then resolved on SDS-PAGE (4–12% gradient gel) under reducing and non-reducing conditions followed with immunoblotting by anti-RAGE antibodies. ns, major non-specific protein species. Monomeric and dimeric forms are marked.

To test whether the observed intermolecular disulfide bonds-sustained RAGE dimers also exist in native cells/tissues, we prepared crude membrane fractions from lung tissues of wild-type and RAGE knockout (KO) mice [23], and resolved the membrane extracts on reducing and non-reducing SDS-PAGE. Murine lung tissues contain two major RAGE isoforms (Figure 3). While the canonical full-length RAGE appears to manifest dimers on non-reducing condition, the fast-migrating RAGE isoform exhibits monomers only in both non-reducing and reducing conditions. Compared to transfected cells, the lung tissue extracts exhibit much less dimers under non-reducing condition, suggesting possible active thiol-disulfide exchanges on the cell surface (see Discussion section).

### RAGE lacking disulfide bridges is retained in the ER

**Figure 4:**
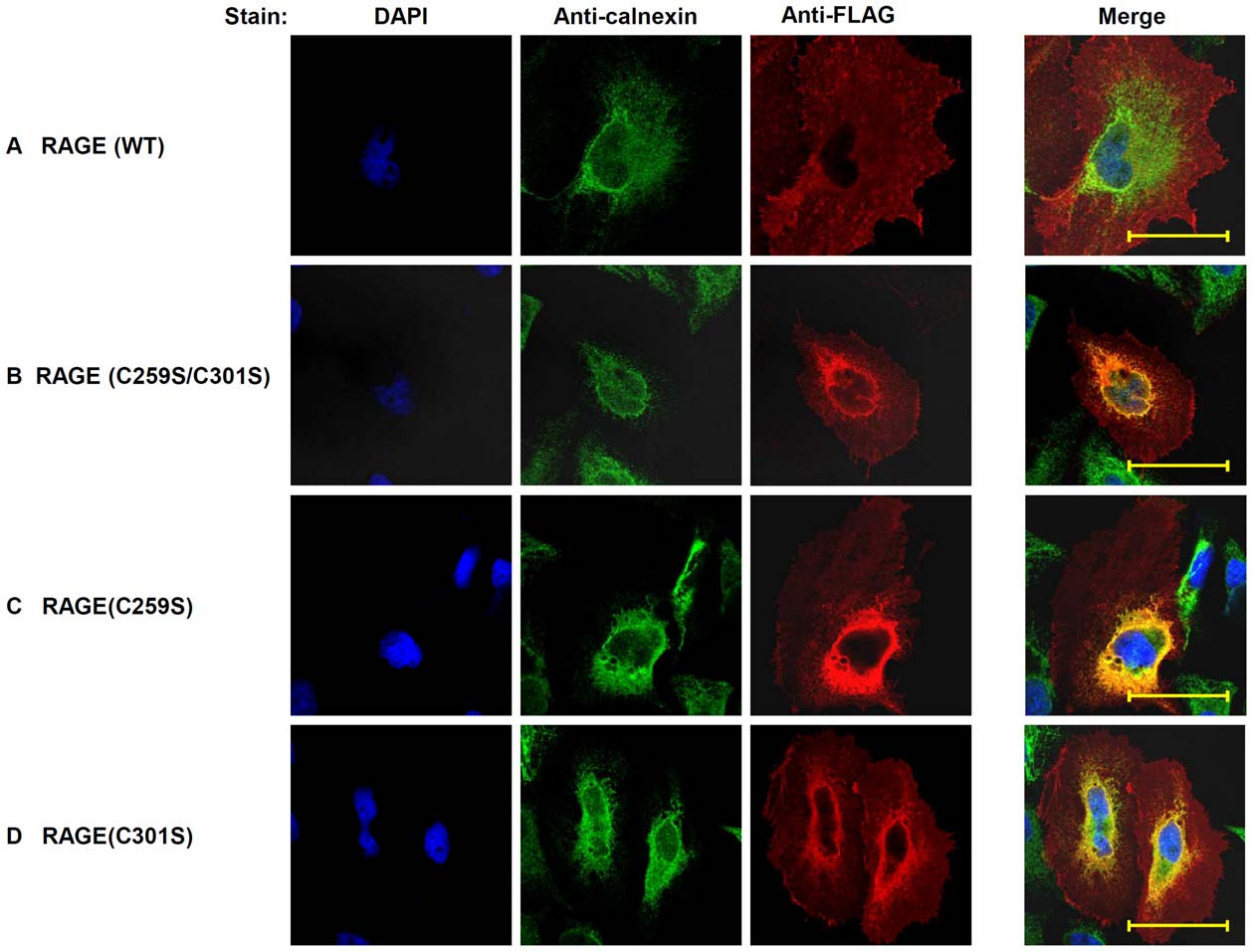
Intracellular localization of RAGE and RAGE cysteine-to-serine mutants. FLAG-tagged RAGE and mutants were transfected to HeLa cells and intracellular immunocytochemistry was performed. Scale bars: 50 µM for all images. (**A**) Localization of RAGE (WT). Blue: DAPI (stain nucleus); green: anti-calnexin (as the ER marker); red: anti-FLAG. Co-localization is demonstrated by the yellow color of the merged image. (**B**) Co-localization of RAGE (C259S/C301S) with the ER marker calnexin. (**C**) Co-localization of RAGE (C259S) with calnexin. (**D**) Co-localization of RAGE (C301S) with calnexin.

Because the formation of disulfide bonds of mammalian membrane proteins occurs in the ER, we next investigated the biological impacts of the disulfide bond-sustained RAGE dimeric structure. We first assessed the subcellular localization of RAGE cysteine-to-serine mutants, *vis-à-vis* the WT counterpart, by immunostaining, and examined the stained cells with confocal microscopy. While RAGE(WT) localizes on the plasma membrane (Figure 4A) [22], RAGE(C259S/C301S) is largely retained within the ER (Figure 4B). This observation propounds the possibility that dimerization of RAGE occurs within the ER, and that the dimeric structure of the receptor on the cell surface is therefore constitutively formed. RAGE carrying single cysteine mutations, RAGE(C259S) and RAGE(C301S) are also retained in the ER (Figure 4, C and D), suggesting that a single intermolecular disulfide bond is not sufficient to sustain a stable RAGE dimer that subsists the cellular quality control system in the ER.

**Figure 5:**
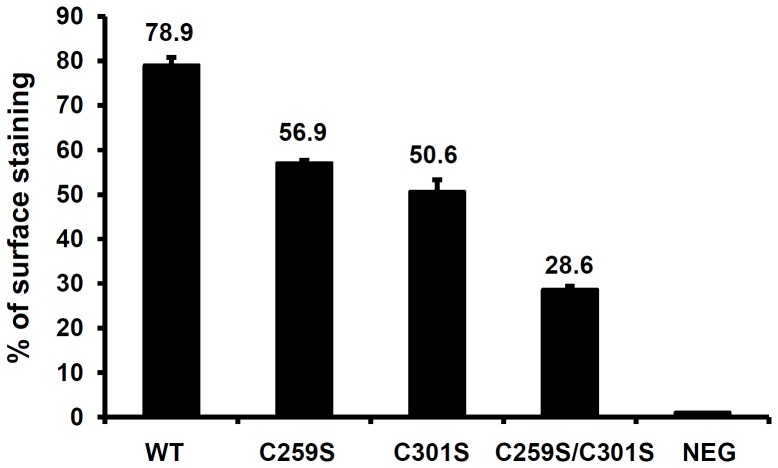
Cell surface expression of RAGE and RAGE cysteine-to-serine mutants. FLAG-tagged RAGE and RAGE mutants were transfected to CHO-CD14 cells. After overnight incubation, the transfected cells (10^6^) were stained with anti-FLAG antibodies and subjected to flow cytometry analyses. Non-transfected cells with same staining were used as negative controls. All values were expressed as mean ± SEM, and the data were from independent transfections (*n* = 3). The *p* value for presented data is <0.01 (ANOVA).

To confirm that the surface expression of RAGE cysteine-to-serine mutants is indeed impaired, we performed cell surface immunostaining, and monitored RAGE cell surface expression in transfected CHO cell populations. While RAGE(C259S/C301S) exhibited significantly lower cell surface expression (28.6%), the percentages of cell surface expression of RAGE(C259S)(56.9%) and RAGE(C301S) (50.6%) were lower than that of RAGE(WT) (78.9%) but higher than RAGE(C259S/C301S) (Figure 5, *p*<0.01), confirming that disablement of the intermolecular disulfide bonds indeed affects RAGE cell surface expression.

### RAGE dimerizes within the ER

**Figure 6:**
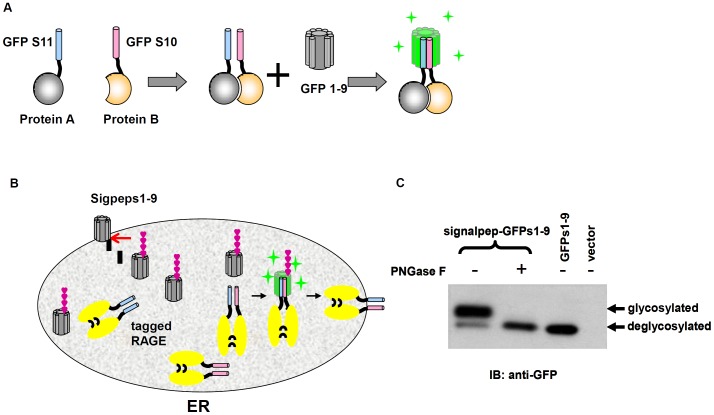
Design of tripartite split GFP complementation to study RAGE dimerization in the ER. (**A**) Illustration of general tripartite split GFP complementation strategy. GFP s10 and s11 are used to tag test proteins whereas GFPs1-9 functions as a detector. When tagged test proteins interact with each other to bring s10 and s11 sufficiently close that they interact with s1-9 to generate green fluorescence. (**B**) Illustration of tripartite split GFP complementation to detect RAGE dimerization in the ER. GFPs1-9 is targeted to the ER with RAGE signal peptide (black bar). Upon entering the ER, the signal peptide is cleaved and GFPs1-9 is glycosylated (magenta chain), and complementation occurs only when s10 and s11-tagged RAGE molecules dimerise. Double disulfide bridge-linked RAGE dimers then leave the ER-Golgi for the cell surface. (**C**) Targeting GFPs1-9 to the ER. Glycosylation of GFPs1-9 confirms that GFPs1-9 is localized in the ER.

To test whether dimers of RAGE are formed within the ER, we employed a split GFP complementation strategy that enables direct observation of protein interactions in the ER [24]. In a tripartite split system, a GFP molecule is split to three parts that consist of two short β strands, s10 and s11, and a large remaining portion, s1-9. The two short strands are used to tag proteins that are subjected to the dimerization test. Unless the two co-expressed, but differentially tagged, proteins interact to bring s10 and s11 tag together to reduce the folding entropy, co-expression of s1-9 will not reconstitute a holoGFP (Waldo and Cabantous, unpublished results, Figure 6A). The working model of tripartite split GFP complementation has been tested with well-studied NF-κB proteins (Supporting information Figure S2).

We modified the complementary system to monitor RAGE dimerization in the ER by fusing GFP s11 and s10 to the NH_2_-terminus of the receptor, and targeting s1-9 into the ER with the signal peptide sequence from RAGE [22]. We reasoned that while GFP s1-9 is targeted to the ER by the signal peptide, as a natively cytosolic protein, this portion of GFP will be temporally retained within the ER rather than being trafficked to the plasma membrane, thus allowing complementation to occur within the ER. Targeting GFP s1-9 to the ER is confirmed by its glycosylation, a modification on N-X-T/S sequence (sequeon) that only occurs in the ER (Figure S1). If RAGE indeed dimerizes within the ER, GFPs1-9 should complement the passing-through s10- and s11-tagged RAGE dimers to generate green fluorescence in this organelle (Figure 6B). This novel approach renders direct observation of RAGE dynamics in the ER in living cells.

**Figure 7:**
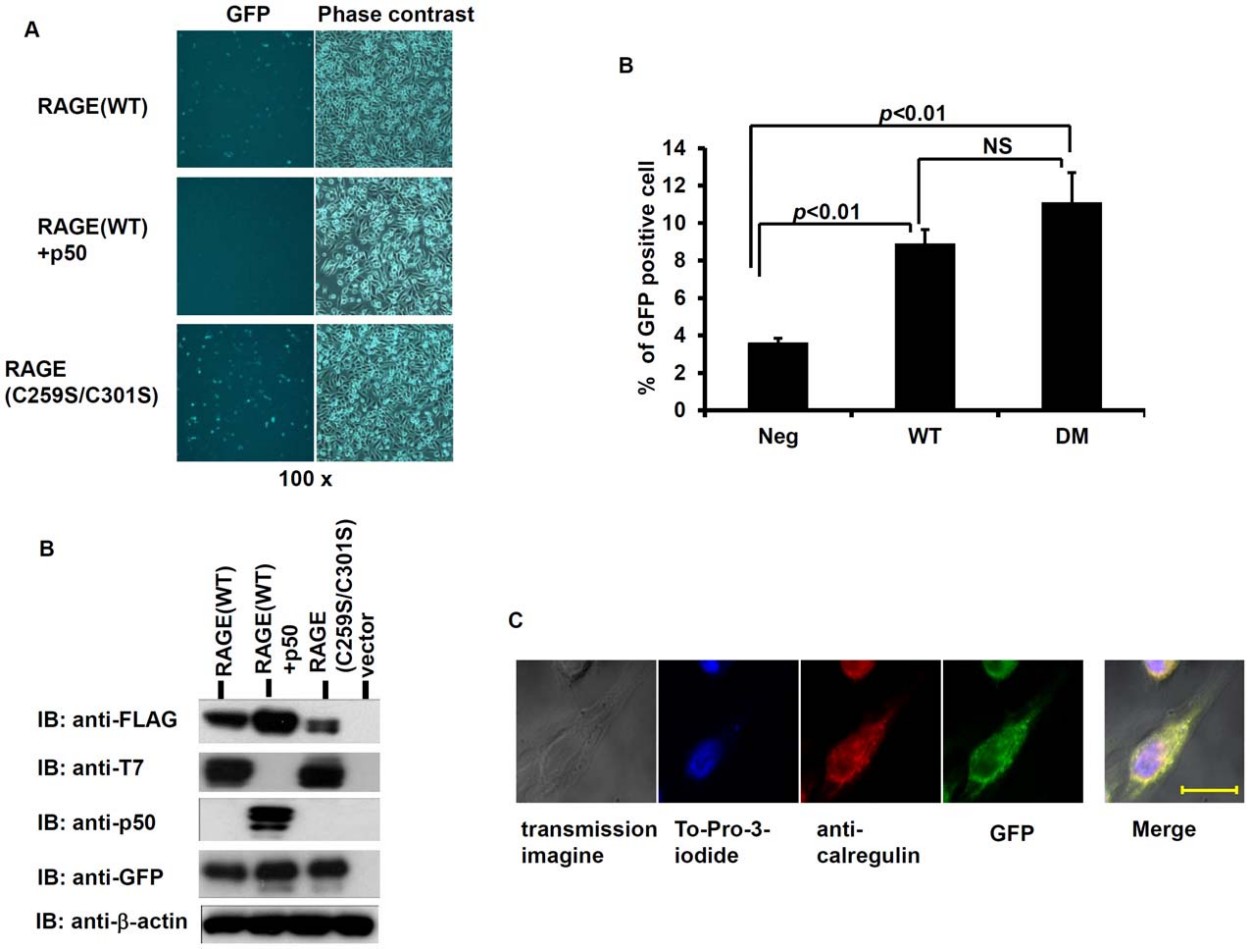
RAGE dimerization in the ER detected by tripartite split GFP complementation (**A**) Tripartite split GFP complementation to detect RAGE dimerization in the ER in CHO-CD14 cell populations. Upper panel: CHO-CD 14 cells transfected with s10-RAGE(WT)-FLAG, s-11-RAGE(WT)-T7, and signalpep-GFPs1-9; middle panel: CHO-CD14 cells tranfected with s10-RAGE(WT)-FLAG, signalpep-s11-p50, and signalpep-GFPs1-9; lower panel: s10-RAGE(C259S/C301S)-FLAG, s11-RAGE(C259S/C301S)-T7, and signalpep-GFPs1-9. (**B**) Immunoblotting of tripartite transfected CHO-CD14 cells to confirm the expression of the individual tripartite components. Unfractionated membrane extracts (7. 5 µg) were resolved with 4–12% NuPAGE gel and anti-β-actin blot was used as the loading control. (**C**) Flow cytometric analysis of reconstituted GFP in transfected cells. Data from 3 independently transfected cells were used (*n* = 3). The GFP positive cells were counted as percentage of the total cells. All values were expressed means ± SEM, and *p*<0.01 (ANOVA); NS, not significant. DM: cysteine 259 and 301 double mutant. (**D**) Localization of reconstituted GFP in the ER observed by confocal microscopy. The wild-type RAGE tripartite components were used, and calregulin was used as the ER marker. To-Pro-3 iodide (642/661) staining was used to mark the nucleus. Scale bar: 50 µm.

CHO-CD14 and HeLa cells were co-transfected with GFP s10- and s11-tagged RAGE, as well as a signalpep-GFPs1-9 construct, and fluorescent and confocal microscopy and flow cytometric analyses were performed. As shown in Figure 7A (upper panel), green fluorescence generated by the tripartite complementation was detected in transfected CHO-CD14 cell populations. To demonstrate that tripartite complementation in the ER is specific, we replaced s11-RAGE with a 365-residue NF-κB p50, a nuclear protein [21] that does not to directly interact with RAGE. s11-p50 is targeted to the ER with the signal peptide from RAGE and the targeting was confirmed by the observation of glycosylation of p50. Co-expression of signalpep-s11-p50, s10-RAGE, and signalpep-GFPs1-9 generate minimal green fluorescence, likely due to stochastic interactions of p50 and RAGE in the ER lumen (Figure 7A, middle panel). The expression of tripartite components was confirmed by IB of the individual component (Figure 7B). Dimerization of RAGE in the ER is further confirmed by co-localization of the reconstituted GFP green fluorescence with the ER marker, calregulin, observed by confocal microscopy (Figure 7D).

In addition to disulfide bonds, non-covalent bonding is intricate of protein interaction. Since the split GFP complementation is highly sensitive, we also used this approach to test whether RAGE(C259S/C301S), which maintains all non-covalent bonds, still forms dimers *in vivo*. As expected, we found that RAGE(C259S/C301S) still forms dimers in the ER (Figure 7A, lower panel), suggesting that non-covalent bonds in RAGE also participate in the formation of dimers. Compared to RAGE(WT), RAGE(C259S/C301S) exhibits similar percentage of GFP positive cells (Figure 7C), although the fluorescence intensity in RAGE(C259S/C301S) transfected cells appears to be stronger, suggesting that the mutant is retained in the ER and thus can reconstitute stable tripartite GFPs in the organelle. These observations imply that RAGE dimers without the double-disulfide bridges in the C2 region are discerned by the quality control system, and retained in the ER (Figure 4).

### The ER quality control system regulates RAGE biogenesis through discerning the intermolecular disulfide bonds

**Figure 8:**
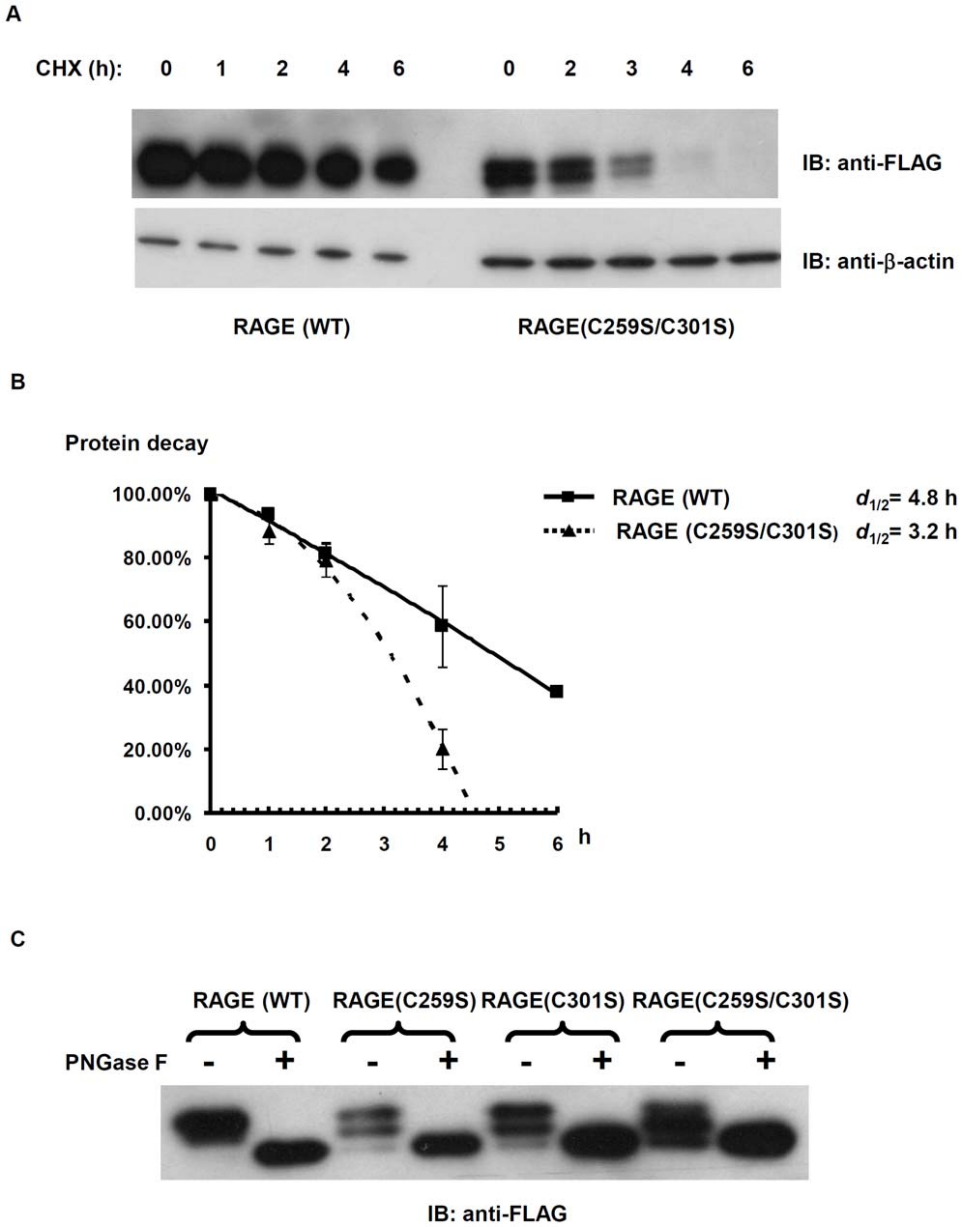
RAGE(C259S/C301S) is unstable and deglycosylated. (**A**) Cyclohaximide chase and IB to compare the protein decay of RAGE(WT) and RAGE(C259S/C301S) in the cells. For FLAG-RAGE transfected cells, 5 µg of lysates were used; whereas for FLAG-RAGE(C259S/C301S) transfceted cells, 10 µg of lysates were used due to the lower expression. After IB with anti-FLAG antibodies, the blot was striped, and reprobed with anti-β-actin antibodies as a loading control. CHX: cyclohaximide. (**B**) Intracellular decay rate of RAGE and RAGE(C259S/C301S) calculated from two CHX chase experiments. The blot intensity was measured with a Kodak Gel Logic 2200 Imaging System and processed with molecular imaging software. The starting point was used as 100% and blot intensity from each time point was calculated relative to the 0 time point. The intensity value of each point was expressed as mean ± SEM, and d_1/2_ was calculated when 50% of the protein I decayed. *C*, RAGE cysteine-to-serine mutants are deglycosylated. Cell lysates from FLAG-tagged RAGE and RAGE cysteine-to-serine mutants were treated with PNGase F, as described, and resolved on a SDS 4–12% NuPAGE gel.

The ER is the major checkpoint of the cellular quality control system for membrane proteins [25], [26]. Improperly folded or unassembled membrane proteins are temporally retained within the ER, either being refolded to continue their journey towards plasma membrane, or exported into the cytoplasm for the ER-associated degradation (ERAD). Membrane, and secreted proteins subjected to the ERAD pathway are deglycosylated and ubiquitinated in the cytoplasm, and then degraded by the proteasome [27]. We observed that the expression level and half-life of RAGE(C259S/C301S) is significantly lower than the WT counterpart (Figure 8, A and B). In addition, the expressed mutated RAGE proteins migrate as triplets on SDS-PAGE (Figure 2D). RAGE contains two sequons for the *N*-linked glycosylation in the extracellular V domain. Treatment of membrane extracts from transfected cells with peptide-*N*-glycosidase F (PNGase F) suggests that the two fast-migrating fragments in RAGE (C259S/C301S) represent singly and doubly deglycosylated RAGE (Figure 8C). Because the mammalian PNGase is located on the cytoplasmic face of the ER [28], [29], deglycosylation of RAGE (C259S/C301S) occurs only after the mutated proteins are exported from the ER to cytoplasm. These observations suggest that unstable RAGE dimers are diverted from the ER into the ERAD pathway.

**Figure 9:**
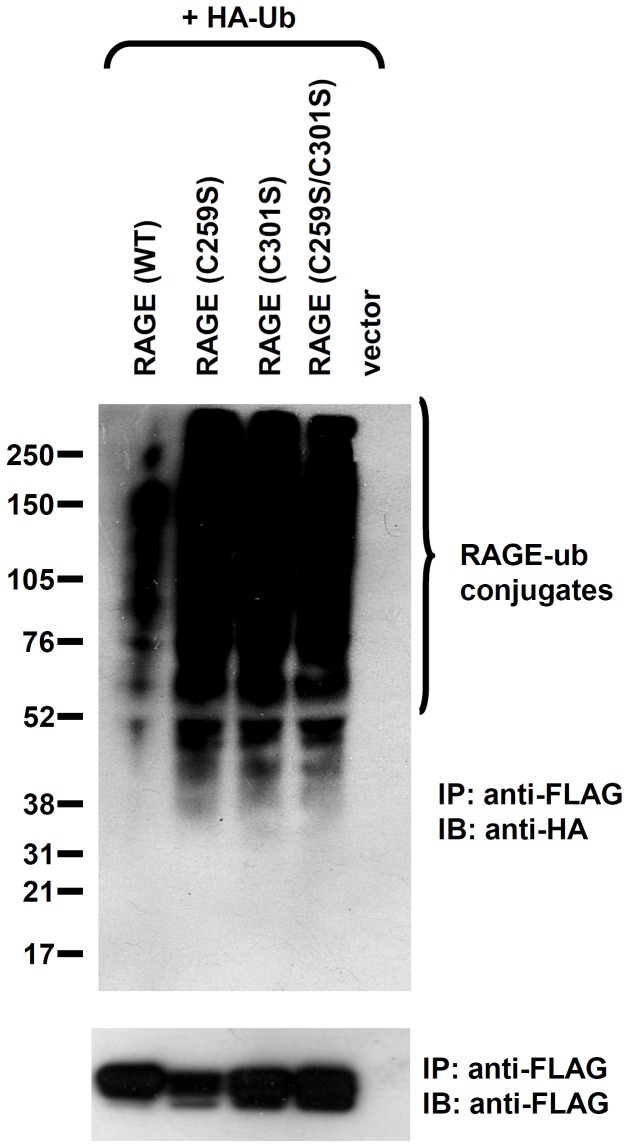
Unstable RAGE dimers are subjected to the ERAD pathway. Ubiquitination assays of RAGE (WT) and RAGE cysteine-to-serine mutants. CHO-CD14 cells were co-transfected with HA-ubiquitin and FLAG-RAGE/RAGE mutants. The transfected cells were then lysed and unfractionated membrane extracts were prepared. The lysates were IPed with anti-FLAG antibodies, and IBed with anti-HA (HRP conjugates) antibodies to demonstrate ubiquitination of RAGE mutants. Anti-FLAG (HRP conjugates) antibodies IB showed the expression of RAGE and RAGE mutants.

Since ERAD substrates are ubiquitinated prior to proteasomal degradation, we examined the ubiquitination status of RAGE cysteine-to-serine mutants and of their WT counterpart. CHO-CD 14 cells were co-transfected with FLAG-tagged RAGE or RAGE cysteine-to serine mutants and an HA-tagged ubiquitin construct, and treated with the proteasome inhibitor MG132 prior to IP. The resolved immunoprecipitants were then blotted with anti-HA and anti-FLAG antibodies. All three RAGE cysteine-to-serine mutants are heavily ubiquitinated, whereas ubiquitination in the WT counterpart is significantly lower under the same condition (Figure 9). These observations demonstrate that unstable RAGE dimers are substrates for the ERAD pathway, and that only stably formed dimeric RAGE molecules are destined for cell surface expression.

## Discussion

Oligomerization of receptors may occur via two basic mechanisms: ligand-induced or constitutive. It has been unclear whether RAGE dimerization needs ligand, and our results showed that RAGE dimerization belongs to the second group (Figures 1 and 7). Using multilateral biochemical approaches, we demonstrated that RAGE in mammalian cells forms dimer-based oligomers (Figure 1). In crosslinking studies, sequential RAGE multimers were detected on SDS-PAGE (Figure 1B), whereas in native PAGE, sRAGE was found to form dimer-based oligomers (Figure 1C). This is due to random crosslinking by the crosslinkers within the oligomeric RAGE complex. Although it is still possible that the observed RAGE oligomers on the cell surface in our studies are formed under an overexpression condition in transfected cells, the relatively short spacer arm (11.4 Å, equal to 8 atoms) of the chosen crosslinker, BS^3^, renders crosslinking among non-complexed RAGE molecules in the crosslinking experiment unlikely. In addition, the expression of RAGE cysteine-to-serine mutants is about 2–4 times lower than the wild-type counterpart (Figures 2D and 8A), whereas the formation of dimers in RAGE carrying single cysteine-to-serine mutation is not affected, suggesting that there is no correlation between the formation of dimers/oligomers and the level of RAGE expression.

While the V and C1 ectodomains of RAGE are directly involved in ligand binding [30], the C2 region regulates the formation of stable dimeric receptors within the ER, and a dimer is likely to be the precursor for the formation of higher-order oligomers (Figure 1C). Zong and colleagues in a recent publication contended that RAGE dimerization is mediated by the V domain and is ligand-dependent [31]. Our data, in contrast, clearly showed that RAGE (ΔV) dimerizes *in vitro* (Figure 2B). To test whether RAGE molecule lacking the V domain still dimerizes *in vivo*, we performed split GFP complementation with RAGE (ΔV). As shown in Supporting information Figure S3A and B, RAGE(ΔV) also forms dimers *in vivo*. In addition, RAGE (ΔV) is similarly expressed on the cell surface as the wild-type and hence is not retained in the ER (Figure S3C). These observations are consistent with a previous observation that a RAGE splice variant containing N-terminal truncation is still expressed on the cell surface [32]. These observations show that the V region is required neither for forming stable dimers, nor for the cell surface expression of RAGE. Of note, Zong and colleagues placed the HA tag at the N-terminus of RAGE signal peptide, such construct may result in RAGE expression without the intended tag, or at abnormal cellular localization, *i.e.* cytoplasm [33].

Oligomerization of cell surface receptors often leads to a change of signal intensity, or induction of complex signaling patterns [34]. It would be expected that oligomerization of RAGE influences either the composition stoichiometry of receptor versus ligand, and/or the ligand binding kinetics, and thus impacts upon downstream signaling events [12]. At present, it is unclear whether the higher-order oligomerization of RAGE occurs within the ER-Golgi apparatus, or on plasma membrane. A recent study of the structure of RAGE V and C1 confirmed our observation that non-covalent bonds also contribute to RAGE dimeric structure (Figure 7A, lower panel), and binding of ligands may enhance or strengthening the oligomeric structure of RAGE on the cell surface [35].

In this study we also developed a novel split GFP complementation approach to monitor RAGE dynamics in the ER (Figures 6 and 7). Relatively low fluorescence intensity and only a small percentage of positive cells were detected in the complementation studies as determined by flow cytometric analyses. This is owing to the fact that only cells transfected by all three plasmids have the possibility to complement, and that only tagged RAGE heterodimers (*i. e.* s10-RAGE and s11-RAGE, see Figure 6B) among tripartite transfected cell populations can emit fluorescence. The percentages of GFP positive cells are similar between RAGE(C259S/C301S) transfected cells and that of RAGE(WT) (Figure 7C). RAGE(C259S/C301S), however, appears to have stronger fluorescence intensity than RAGE (WT) (Figure 7A). Given that both mutant and the WT had similar percentage of GFP positive cells, the observed stronger fluorescence intensity is likely due to that RAGE(C259S/C301S) proteins are retained within the ER (Figure 4B), allowing formation of more stable GFP complexes on-site, whereas RAGE(WT) proteins in the ER are constantly trafficked to the apical plasma membrane so that their tripartite complementation in the ER is of a more temporary nature.

Structural studies on soluble RAGE did not find intermolecular disulfide bridges in the C2 region [30], [36]. However, sRAGE used in these studies was expressed in *E. coli* host, whereas protein disulfide isomerases that regulate the formation of disulfide bonds reside in the ER of mammalian cells. An early report demonstrated via peptide sequencing of sRAGE purified from mouse lung tissues that the two cysteines in the C2 region form an intramolecular disulfide bridge [37]. We also found that in mouse lung where majority of detected RAGE appears to be in monomeric form under non-reducing conditions (Figure 3). Because the lung has a large surface directly exposed to high levels of oxygen and reactive reagents [38], [39], we suspect that the observed non-disulfide bond-sustained species in the lung a result of thiol-disulfide exchanges on the lung cell surface. It has been reported that CD4 on the cell surface exhibits active thiol-disulfide exchanges, and such exchange regulates the entry of HIV-1 viruses to T cells [40]. Similar thiol-disulfide exchange on the cell surface has also been observed in CD30 [41]. RAGE is expressed in diverse cell type and tissues, and hence exposed to vastly different physiological environment. It is possible that, depending upon the microenvironment, the intermolecular disulfide-bridge sustained RAGE observed in transfected cell system becomes the major cell surface species. Currently, because the available anti-RAGE antibodies do not IP RAGE, we are unable to assess the status and ratio of the RAGE species in other native cell types and tissues. The mixed RAGE isoforms in transfected cells also precludes us from direct assessing the signaling specificity or capacity of the intermolecular disulfide bridge-linked RAGE dimers/oligomers. Future efforts including identifying the regulatory elements that contribute to RAGE cell surface configuration should help to resolve these issues.

The double disulfide-linked dimeric structure of RAGE appears to serve at least one clear purpose: it functions as an inherent structural hallmark that is discerned by the ER quality control system, and hence plays a critical role in RAGE biogenesis. In transfected cells, a portion of expressed RAGE carrying cysteine-to-serine mutations still reaches the cell surface (Figure 4 and 5). This is likely due to the overexpression of the mutant proteins that overwhelms the ER, thus allowing a portion of the mutant proteins to be spared from the ERAD. In nature, unassembled RAGE monomers, or dimers un-bridged by the double disulfide bonds within the ER are expected either to be refolded and reassembled with the aid of ER chaperones, or to be diverted *en route* to the ERAD pathway for their complete destruction. Despite retaining the capacity to form oligomers *in vitro*, RAGE carrying single cysteine mutations, C259S or C301S, behave similarly as RAGE(C259S/C301S) *in vivo* with respect to ER retention (Figure 4B) and ubquitination status (Figure 9), manifesting the stringency of the ER cellular quality control system during the biogenesis of RAGE. Although undistinguishable in the ER retention and ubiquitination status, RAGE molecules carrying single and double cysteine mutations clearly showed a different level of cell surface expression (Figure 5), confirming that the formation of the intermolecular disulfide bonds in the C2 region is clearly correlated with the cell surface expression of the receptor.

While RAGE dimerization is constitutive, our discovery of RAGE homo dimerization via intermolecular disulfide bonds raises an interesting and intriguing possibility that the state of extracellular reduction-oxidation reactions (redox) may regulate RAGE signaling by altering the receptor's oligomeric status on the cell surface. Redox regulate diverse biological processes including enzymatic reactions, gene expression, and signaling events [42]. A change of redox state generates cellular inflammation and contributes to the development of chronic diseases [43]–[45]. Although the formation of C_259_ and C_301_ intermolecular covalent links is critical for the biogenesis of RAGE, such fail-safe function may be forfeited once the receptor reaches cell surface. Instead, oscillation of physiological redox state in vascular and nervous system as well as in extracellular milieu, or regulation by extracellular thiol-isomerases, which are often activated by stress conditions, may facilitate thiol-disulfide exchanges, leading to the shift of the oligomeric status or disulfide boding profile of RAGE on the cell surface. This new layer of regulation may activate or negate RAGE signaling, or generate new RAGE signaling patterns.

## Supporting Information

Figure S1 **Disulfide bond-mediated dimer formation in HeLa cells.** Left panel, RAGE(WT) and deletion mutants in non-reducing SDS-PAGE; right panel, RAGE(WT) and deletion mutants in reducing SDS-PAGE.
(TIF)

Figure S2 **Test tripartite split GFP complementation with NF-κB proteins.** The well-studied NF-κB proteins were used as the working model to test tripartite split GFP complementation in the cell. NF-κB p65(RelA) is known to form stable complex with the inhibitor IκBα in the cell, and IκBα does not form homodimers. (**A**) p65 and IκBα complex as a model to demonstrate tripartite split GFP complementation in the cell. GFPs10 tagged p65, GFPs11tagged IκBα, and detector GFPs1-9 were co-transfected to CHO-CD14 cells. After overnight incubation, fluorescence microscopy was conducted to monitor GFP complementation in cell population. (**B**) Negative control. GFPs10 and s11 tagged IκBα and GFPs1-9 were transfected to CHO-CD14 cells, and fluorescence generated in this setting is the background due to stochastic interactions of IκBα. (**C**) Western blotting to verify the expression of the tripartite components.
(TIF)

Figure S3 **RAGE lacking the V region dimerises in the ER and is expressed on the cell surface.** (**A**) Tripartite split GFP complementation assays with s10- and s11-tagged RAGE(ΔV) and signalpepGFPs1-9 in CHO-CD14 cells. RAGE(WT) tripartite transfection was used as the positive control, and s10-RAGE(WT) plus signalpep s11-p50 and signalpepGFPs1-9 tripartite transfection serves as the negative control. (**B**), Flow cytometric analysis of reconstituted GFP in transfected cells. Data from 3 independently transfected cells were used (*n* = 3). The GFP positive cells were counted as percentage of the total cells. All values were expressed means ± SEM, and *p*<0.05. (**C**), Confocal microscopy of FLAG-RAGE(ΔV) transfected HeLa cells demonstrates that RAGE (ΔV) does not co-localize with the ER marker. Intracellular immunostaining was performed with calnexin as the ER marker, and DAPI staining marks the nucleus. Scale bar: 50 µm.
(TIF)
