# Supplementary figures and images for: Disulfide Bonds within the C2 Domain of RAGE Play Key Roles in Its Dimerization and Biogenesis
Source: PLoS One. 2012 Dec 17;7(12):e50736. doi: 10.1371/journal.pone.0050736 (PMC3524233; doi:10.1371/journal.pone.0050736)

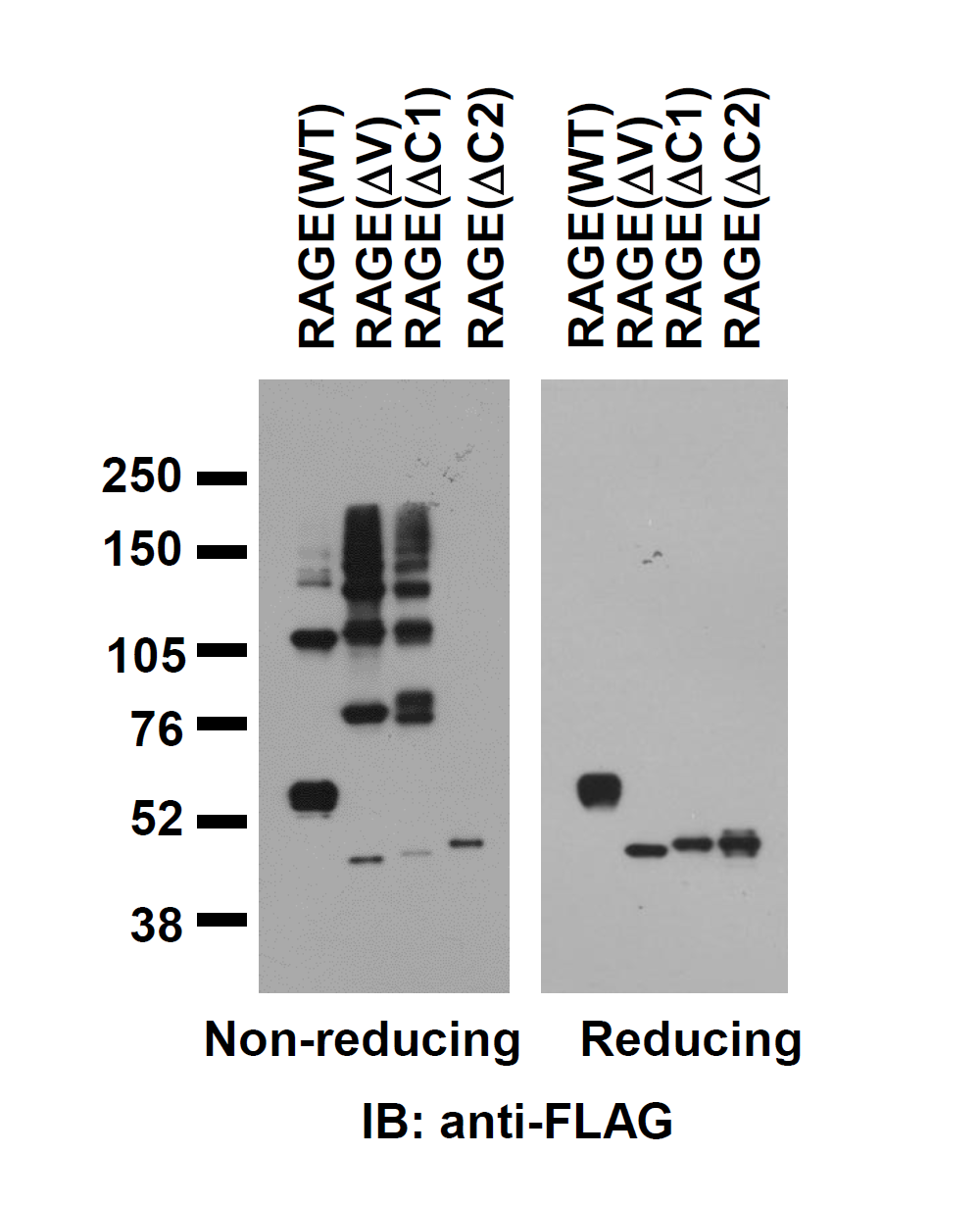

Supplement: Figure S1 — Disulfide bond-mediated dimer formation in HeLa cells. Left panel, RAGE(WT) and deletion mutants in non-reducing SDS-PAGE; right panel, RAGE(WT) and deletion mutants in reducing SDS-PAGE. (TIF) [file pone.0050736.s001.tif]

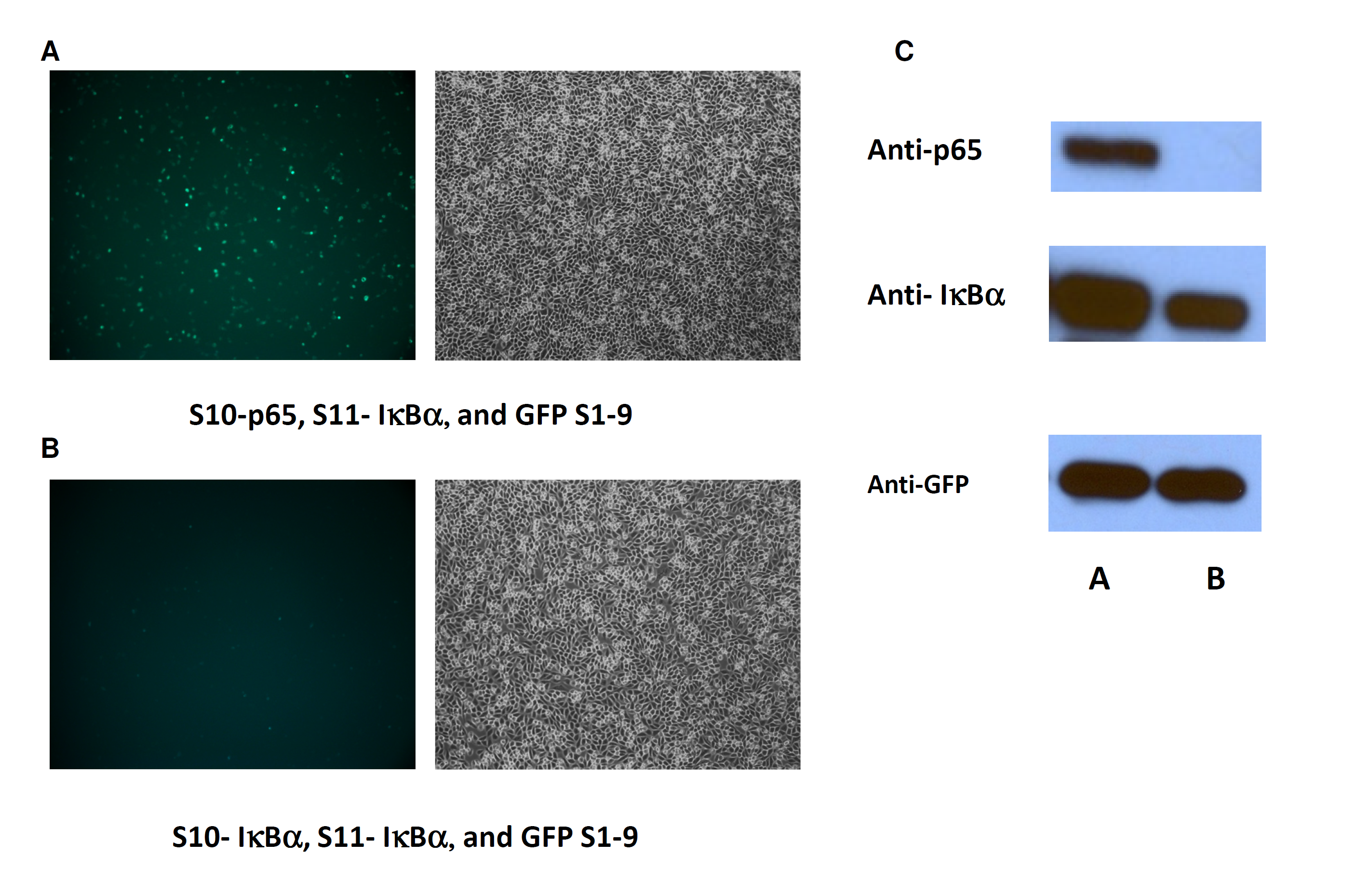

Supplement: Figure S2 — Test tripartite split GFP complementation with NF-κB proteins. The well-studied NF-κB proteins were used as the working model to test tripartite split GFP complementation in the cell. NF-κB p65(RelA) is known to form stable complex with the inhibitor IκBα in the cell, and IκBα does not form homodimers. (A) p65 and IκBα complex as a model to demonstrate tripartite split GFP complementation in the cell. GFPs10 tagged p65, GFPs11tagged IκBα, and detector GFPs1-9 were co-transfected to CHO-CD14 cells. After overnight incubation, fluorescence microscopy was conducted to monitor GFP complementation in cell population. (B) Negative control. GFPs10 and s11 tagged IκBα and GFPs1-9 were transfected to CHO-CD14 cells, and fluorescence generated in this setting is the background due to stochastic interactions of IκBα. (C) Western blotting to verify the expression of the tripartite components. (TIF) [file pone.0050736.s002.tif]

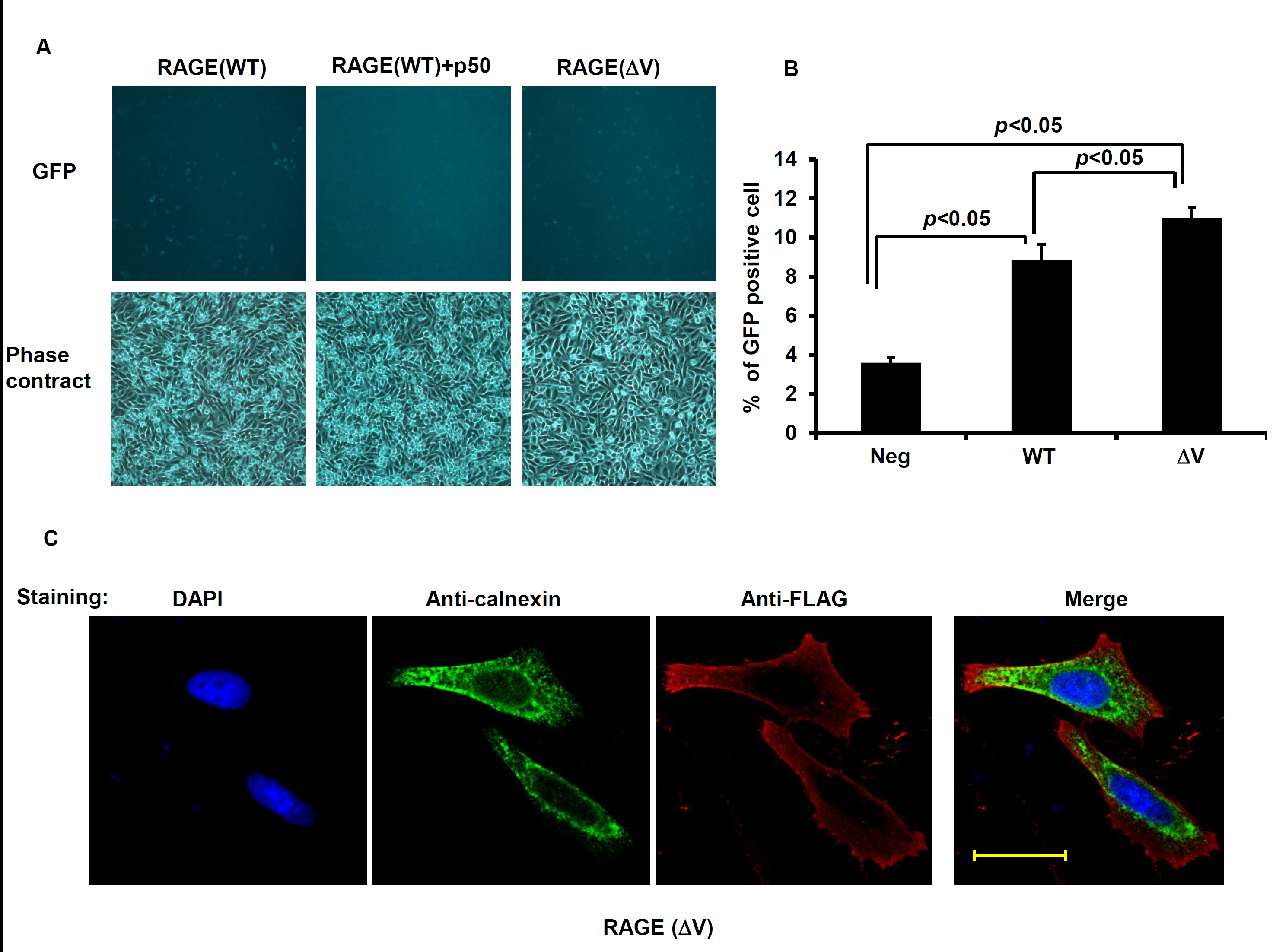

Supplement: Figure S3 — RAGE lacking the V region dimerises in the ER and is expressed on the cell surface. (A) Tripartite split GFP complementation assays with s10- and s11-tagged RAGE(ΔV) and signalpepGFPs1-9 in CHO-CD14 cells. RAGE(WT) tripartite transfection was used as the positive control, and s10-RAGE(WT) plus signalpep s11-p50 and signalpepGFPs1-9 tripartite transfection serves as the negative control. (B), Flow cytometric analysis of reconstituted GFP in transfected cells. Data from 3 independently transfected cells were used (n = 3). The GFP positive cells were counted as percentage of the total cells. All values were expressed means ± SEM, and p<0.05. (C), Confocal microscopy of FLAG-RAGE(ΔV) transfected HeLa cells demonstrates that RAGE (ΔV) does not co-localize with the ER marker. Intracellular immunostaining was performed with calnexin as the ER marker, and DAPI staining marks the nucleus. Scale bar: 50 µm. (TIF) [file pone.0050736.s003.tif]
